# AMPK–NLRP3 Inflammasome Crosstalk: Structural Insights, Molecular Mechanisms, and Therapeutic Implications for Inflammation and Neuroinflammation

**DOI:** 10.1007/s12035-026-05932-7

**Published:** 2026-06-09

**Authors:** Triveni kodi, Krishnadas Nandakumar, Anoop Kishore

**Affiliations:** Department of Pharmacology, Manipal College of Pharmaceutical Sciences, Manipal Academy of Higher Education, Manipal, India

**Keywords:** AMPK, NLRP3 inflammasome, Autophagy, Sirtuins, Alzheimer’s disease, Neuroinflammation

## Abstract

The nucleotide-binding domain leucine-rich repeat and pyrin domain-containing protein-3 (NLRP3) inflammasome plays a central role in inflammatory diseases, including cardiovascular, gastrointestinal, neurodegenerative, autoimmune, hepatic, renal, and pulmonary disorders. Although various cellular pathways tightly regulate its activation, the precise mechanisms remain unclear. Emerging evidence highlights adenosine monophosphate-activated protein kinase (AMPK) as a critical regulator of energy balance and cellular metabolism, suggesting its potential involvement in modulating NLRP3 inflammasome activity. This review explores the structural dynamics of the NLRP3 inflammasome and the activation of AMPK signaling. It focuses on the mechanistic pathways underlying AMPK-mediated suppression of the NLRP3 inflammasome, including autophagy-dependent regulation, sirtuin-mediated regulation, ER stress/TXNIP signaling pathway, regulation of mitochondrial homeostasis, NF-κB/MyD88-mediated priming, and maintenance of lysosomal integrity. Furthermore, the review discusses the interplay of these pathways in CNS-specific disease models, such as Ischemic stroke/cerebral ischemia, Alzheimer’s disease, Parkinson’s disease, multiple sclerosis, diabetic neuropathy, and neuroinflammation models, as well as traumatic brain injury, streptozotocin-induced neuroinflammation, and lipopolysaccharide-induced neuroinflammation. Additionally, this review examined therapeutic strategies targeting the AMPK–NLRP3 axis in neuroinflammatory and neurodegenerative disorders.

## Introduction

Inflammasomes are intracellular multiprotein complexes within immune cells that become activated during certain inflammatory responses. Upon activation, these proteins further aggravate the inflammatory processes. Among these complexes, the nucleotide-binding domain leucine-rich repeat and pyrin domain-containing protein-3 (NLRP3) inflammasome has been extensively studied due to its involvement in several pathological conditions, including Parkinson’s disease, Alzheimer’s disease, acute liver injury, myocardial injury, and ulcerative colitis [1–5]. NLRP3 functions as an intracellular innate immune sensor, detecting danger signals and triggering inflammatory responses [6]. It is primarily expressed in macrophages, but it is also present in other immune and non-immune cells, including granulocytes, dendritic cells, monocytes, T and B lymphocytes, epithelial cells, and osteoblasts [7–9].

In the central nervous system (CNS), persistent cellular stress activates the NLRP3 inflammasome, amplifying the inflammatory cascade and potentially altering blood–brain barrier (BBB) permeability. This may facilitate the entry of peripheral immune cells and inflammatory mediators into the CNS [10, 11]. In the periphery, NLRP3 activation triggers systemic inflammation, elevating IL-1β and IL-18 levels. The resulting disruption of the BBB allows immune infiltration and further amplifies neuroinflammation [12]. Overactivation of microglia and astrocytes propagates CNS inflammation, ultimately leading to neuronal apoptosis and neurodegeneration [13]. The activated NLRP3 inflammasome aids in the maturation and secretion of proinflammatory cytokines, contributing to the body’s immune defenses as well as to the progression of chronic inflammatory disorders [14, 15]. Owing to its widespread expression and crucial role in inflammation, NLRP3 has attracted attention as a target for regulating several inflammatory and neurological disorders. However, despite extensive research, NLRP3 inhibitors have not successfully entered clinical practice.

Phosphorylated AMPK plays an important role in regulating NLRP3 activation. AMPK is a cellular energy sensor that becomes activated during low-energy conditions. Activation of AMPK through phosphorylation maintains cellular energy homeostasis and restores metabolic functions. Activated AMPK is known to suppress the NLRP3 inflammasome via several mechanisms, including enhanced autophagy that facilitates the clearance of damaged cells, sirtuin activity, mitigation of mitochondrial dysfunction, amelioration of ER stress, prevention of lysosomal disruption, and suppression of the myddosome complex [16–20]. Therefore, the AMPK signaling pathway could serve as a promising target for regulating NLRP3 inflammasome-driven diseases, including neuroinflammation, metabolic disorders, and autoimmune diseases.

In this review, we present an overview of the structural dynamics of AMPK and NLRP3 activation, as well as the mechanistic pathways underlying AMPK-mediated suppression of the NLRP3 inflammasome and their interplay in CNS-specific disease models. Additionally, we discuss the therapeutic strategies targeting the AMPK–NLRP3 axis in neuroinflammatory and neurodegenerative disorders.

## Structural Dynamics of NLRP3 Inflammasome and AMPK

**Fig. 1:**
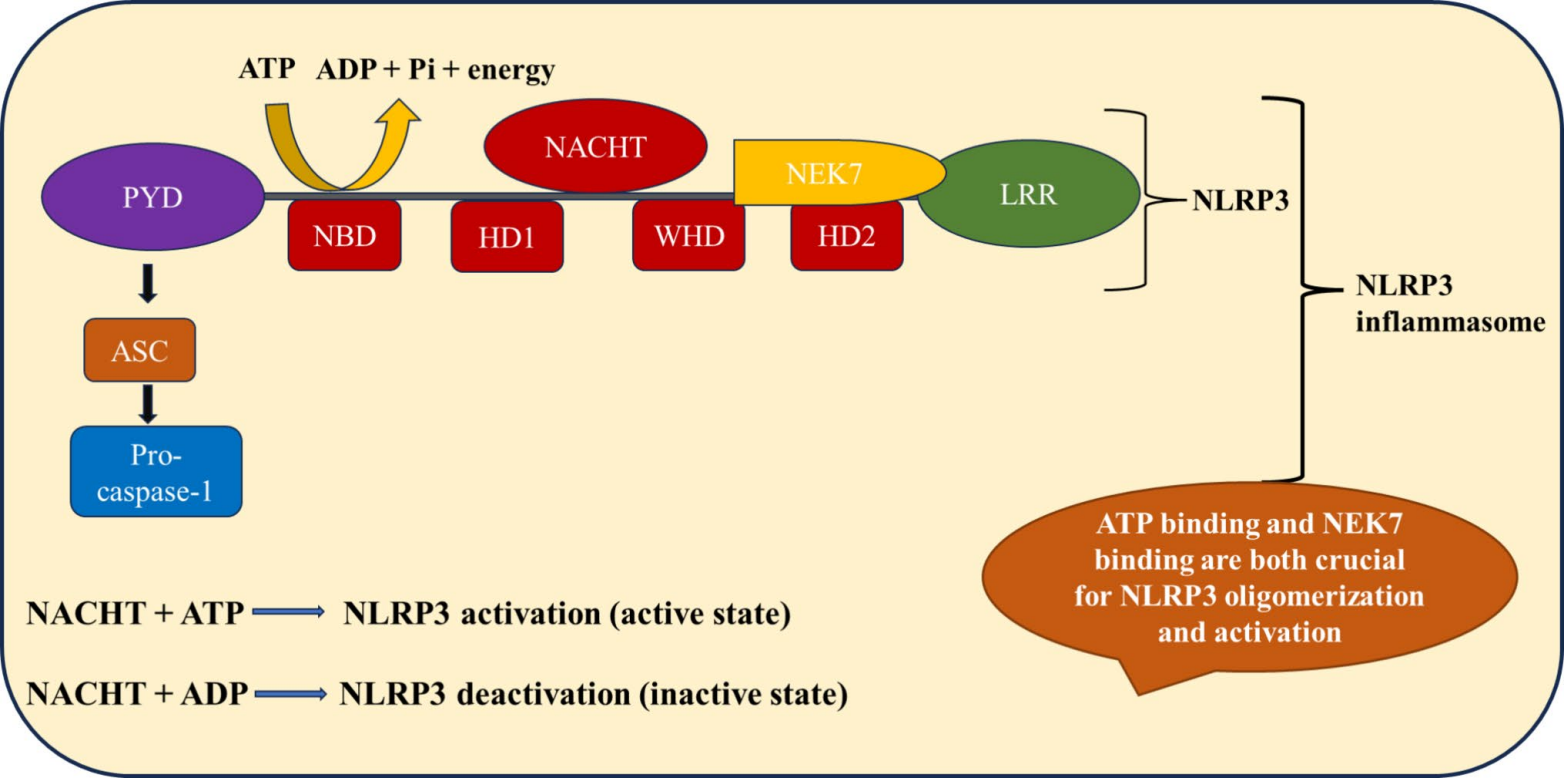
Structural organization and dynamic activation of the NLRP3 inflammasome. NEK7 binding promotes structural rearrangement and oligomerization of NLRP3 into its active form. Subsequently, the N-terminal PYD domain of NLRP3 recruits ASC and pro-caspase-1, leading to inflammasome assembly. NLRP3, nucleotide-binding domain leucin-rich repeat and pyrin domain-containing protein-3; NACHT, nucleotide-binding oligomerization domain; PYD, pyrin domain; NBD, nucleotide-binding domain; HD1, helical domain 1; WHD, winged helix domain; HD2, helical domain 2; LRR, leucine-rich repeat; ASC, apoptosis-associated speck-like protein; CARD, caspase-recruitment domain

The NLRP3 inflammasome is composed of three components: the cytosolic sensor protein NLRP3, the adaptor protein apoptosis-associated speck-like protein (ASC), and the cysteine protease pro-caspase-1. The NLRP3 sensor protein is a tripartite protein that contains an N-terminal pyrin domain (PYD), a central nucleotide-binding and oligomerization domain (NACHT), and a C-terminal leucine-rich repeat (LRR) [21]. Under normal conditions, the LRR domain maintains autoinhibition and keeps NLRP3 in an inactive state. In addition, the LRR domain responds to endogenous signals and contributes to conformational changes (transition from inactive to active state) required for inflammasome activation. Upon activation of NLRP3, the N-terminal PYD domain recruits the inflammasome components ASC and pro-caspase-1 [22–24]. The NACHT domain is further divided into four sub-domains: the nucleotide-binding oligomerization domain (NBD), helical domain 1 (HD1), winged helix domain (WHD), and helical domain 2 (HD2). It also contains signal transduction ATPases with numerous domains (STAND) elements, such as Walker A, Sensor 1, and Walker B motifs [25]. Among these, Walker A enables ATP binding, while Walker B is responsible for ATP hydrolysis, both of which are essential for NLRP3 inflammasome activation. ATP binding to the Walker A motif of the NACHT domain activates NLRP3 and initiates ATP hydrolysis, converting ATP to ADP and releasing a phosphate group along with energy. This catalyzes conformational changes in NLRP3, aiding the recruitment of NEK7. NEK7 functions upstream of the NLRP3 inflammasome [26]. The binding of NEK7 facilitates structural rearrangement and oligomerization of NLRP3, transitioning it into an active conformation [25, 27]. The oligomerized NLRP3 then recruits ASC through PYD–PYD interactions, forming the ASC speck. Subsequently, ASC interacts with pro-caspase-1 via CARD–CARD interactions, leading to the activation of caspase-1. Activated caspase-1 cleaves pro-IL-1β and pro-IL-18 into their active forms (IL-1β and IL-18), which amplify the inflammatory response (Fig. 1). This cascade contributes to the body’s immune defense systems but can also drive pathological inflammation when unregulated.

ADP bound to the NACHT domain of NLRP3 exists in an inactive state, characterized by a closed and autoinhibited conformation. When ADP is converted to ATP in response to activation signals such as pathogen-associated molecular patterns (PAMPs) and damage-associated molecular patterns (DAMPs), the resulting conformational changes promote NLRP3 activation, oligomerization, and inflammasome assembly. The balance between inactive ADP- and active ATP-bound states of the NACHT domain is crucial for regulating inflammasome activity. This regulation ensures that inflammation is initiated only in response to pathological signals [25, 27]. Owing to its involvement in various inflammatory disorders, the NLRP3 inflammasome represents a promising target for drug discovery.

AMPK exists as a heterotrimeric serine/threonine protein kinase, composed of three subunits α (catalytic), β (scaffolding), and γ (regulatory) in a 1:1:1 proportion [28–30]. The α-subunit contains pivotal residue Thr172 at the kinase domain (KD), which is phosphorylated by upstream kinases such as liver kinase B1 (LKB1) [31], calcium/calmodulin-dependent protein kinase kinase (CaMKK β) [32], and transforming growth factor β-activated kinase 1 (TAK1) [33]. Additionally, the KD is bound by the autoinhibitory domain (AID), which limits its mobility and prevents kinase activity in the absence of AMP, while the α-liner connects the AID and c-terminal domain (CTD) of the α-subunit. The α-CTD connects the β- and γ-subunits [34, 35]. The β-subunit N-terminal is myristoylated and contains Ser108 at the carbohydrate-binding module (CBM), where glycogen interacts, while the C-terminal domain of the β-subunit stabilizes the interaction between α and β. Many direct inhibitors that cause allosteric activation bind to the allosteric drug and metabolite (ADaM) site at the KD and CBM interface [36, 37].

The γ-subunit of AMPK contains four consecutive cystathionine-β-synthase (CBS) domains and provides four AMP-binding sites (sites 1–4). Sites 1 and 3 can bind transiently, while site 4 binds tightly to AMP; site 2 does not contain an AMP-binding domain. Site 1 has a higher binding affinity for AMP than site 3 [30, 35]. An enhanced cellular AMP/ATP ratio allows AMP to bind to site 1, a process known as allosteric activation of AMPK. AMP binding to site 3 induces conformational changes in the α-subunit and allows the α-linker to bind to the γ-subunit, pulling the AID away from the KD [38]. This enhances the accessibility of Thr172 for phosphorylation by upstream kinases and protects it from dephosphorylation by protein phosphatases [28, 39].

Once AMPK is activated, it enhances the catabolic pathways that generate ATP, such as glycolysis, lipolysis, β-oxidation, and glucose uptake, while inhibiting anabolic pathways that consume ATP, including fatty acid synthesis, protein synthesis, and gluconeogenesis. Direct AMPK activators such as A-769662, PT1, salicylate, and compound 991 bind to the ADaM site, resulting in allosteric activation and a fivefold increase in kinase activity. In contrast, indirect activation of AMPK by AICAR, metformin, resveratrol, quercetin, epigallocatechin, and related compounds leads to Thr172 phosphorylation at the α-subunit, enhancing kinase activity by up to 100-fold. Consequently, phosphorylation at Thr-172 of the α-subunit is generally considered an on–off switch for AMPK [37].

## Mechanisms of AMPK-Mediated Suppression of the NLRP3 Inflammasome

Activated AMPK regulates the NLRP3 inflammasome in several inflammatory and neuroinflammatory conditions. Although the current understanding of how AMPK controls NLRP3 activation remains limited, several indirect mechanisms have been identified through which activated AMPK suppresses the inflammasome. These include AMPK-mediated enhancement of autophagy, stimulation of SIRT1, mitigation of ER stress, reduction of mitochondrial dysfunction, limitation of lysosomal disruption, and suppression of the myddosome complex [20, 40]. The following sections provide an overview of these processes.

### Autophagy-Dependent Regulation

Autophagy is a lysosome-dependent self-degradative process that removes intracytoplasmic misfolded proteins, dysfunctional organelles, and other cellular debris. Dysregulation of autophagy results in the accumulation of aggregated proteins and increases their toxicity [41–43]. Autophagy is typically triggered under physiologically stressful conditions such as starvation and oxidative stress [44, 45]. It is regulated by several genes, including autophagy-related 5 (ATG5) protein, microtubule-associated protein 1light chain 3 (LC3), Beclin-1 (BECN-1), ULK1 (Unc-51-like kinase 1, a mammalian ortholog of Atg1), tuberous sclerosis protein 2 (TSC2), autophagy-related 9 (ATG9) protein, phosphatidylinositol 3-kinase catalytic subunit type 3 (PI3KC3), receptor of activated C kinase 1 (RACK1), and mammalian target of rapamycin (mTOR) [44, 46–48].

AMPK enhances autophagy by phosphorylating autophagy-related proteins at various stages of the process [46]. Evidence suggests that AMPK induces autophagy through direct phosphorylation and activation of ULK1 [49–53], PI3KC3 [54, 55], BECN-1 [56, 57], and ATG9 [57, 58], as well as through indirect inhibition of mTOR [59–61] via phosphorylation of TSC1/TSC2 [62, 63]. The crosstalk between autophagy and inflammasomes is essential for maintaining cellular homeostasis [42, 64, 65].

Active autophagy plays a critical role in regulating the NLRP3 inflammasome by suppressing endogenous signals and degrading inflammasome components [20, 66]. This regulatory effect is closely linked to inhibition of mTOR signaling, which activates autophagy and ultimately contributes to suppression of NLRP3 activation [64, 67, 68]. Similarly, dysfunction of autophagy results in activation of the NLRP3 inflammasome [20, 42]. A negative feedback loop involving autophagy and inflammasomes has also been observed: activated inflammasomes trigger enhanced autophagy, which in turn limits inflammasome function [42, 69]. This interplay is particularly relevant in neurodegenerative disorders, where defective autophagy contributes to sustained NLRP3-driven neuroinflammation.

Autophagy degrades inflammasome components and limits NLRP3 activation [66, 70]. Several preclinical studies support this mechanism. For example, autophagy clears mtDNA and mtROS during ischemic reperfusion injury in the brain, thereby blocking NLRP3 inflammasome activation and IL-1β expression [71]. Pretreatment with the AMPK activator metformin during focal cerebral ischemia reduced infarct volume, neurological deficits, and cell apoptosis by augmenting AMPK phosphorylation and autophagy activation in the rat brain [72]. In a model of asphyxia-induced cardiac arrest, metformin pretreatment decreased neuronal degeneration and glial activation in the rat cortex and hippocampal CA1 regions via AMPK-activated autophagy stimulation [73]. In a rat burn wound model, activation of autophagy reduced wound progression by inhibiting NLRP3 inflammasome activity [74]. Metformin also promoted M2 macrophage polarization and suppressed NLRP3 inflammasome activation through the AMPK/mTOR pathway, thereby enhancing wound healing [75].

Autophagy is reduced during aging, and enhancing autophagy has been shown to extend lifespan from worms to mammals by suppressing the NLRP3 inflammasome complex [20, 76]. Rapamycin, an autophagy inducer, prolongs life span by inhibiting mTOR [20, 77] and reducing mitochondrial ROS [78]. Metformin inhibits mTOR and promotes autophagy [79]. These effects can be blocked by the autophagy inhibitor 3-MA [80, 81]. Similarly, 3-MA reverses resveratrol-mediated suppression of the NLRP3 inflammasome in J774A.1 macrophages [70, 82].

Numerous studies demonstrate that AMPK plays a key role in enhancing autophagy. Reduced AMPK levels diminished adiponectin’s protective effects in HepG2 cells, leading to ethanol-induced apoptosis. Adiponectin has been shown to inhibit inflammasome activation and promote autophagy via AMPK signaling [44, 83]. AMPK knockdown inhibited resveratrol-induced autophagy in leukemia cells [84]. Impairment of autophagy function is associated with numerous diseases, including aging, cancer, inflammation, infectious diseases, and neurodegenerative disorders [85]. Furthermore, autophagy contributes to decreased cell mortality, increased myelination, and improved recovery of motor function [81].

### Sirtuin-Mediated Regulation

The sirtuin family consists of seven proteins (SIRT1–SIRT7), characterized by NAD⁺-dependent deacetylase and ADP-ribosyl transferase activity. Although they share a conserved core domain, they differ in their N- and C-terminal regions, cellular localization, and biological functions [20, 86]. SIRT1, SIRT6, and SIRT7 are located in the nucleus; SIRT3, SIRT4, and SIRT5 are mitochondrial proteins; and SIRT2 is cytoplasmic [87–89]. The expression and functions of sirtuins vary according to isoform and tissue type, and they are expressed in both the central nervous system and peripheral tissues [90]. Sirtuins regulate diverse cellular processes, including mitochondrial biogenesis, autophagy, apoptosis, inflammation, oxidative stress, ER stress, and insulin sensitivity [86, 91].

SIRT1 functions mainly by removing acetyl groups from target proteins, thereby influencing their activity, stability, and interactions. It plays a key role in neuroprotective mechanisms, including neurogenesis, neuronal survival [92], and cognition [71, 93–95]. SIRT1 has also been extensively studied in the context of aging. Owing to their anti-aging activity, sirtuins are often referred to as the “enzymes of youth” [96].

AMPK and SIRT1 share similar functions in cellular survival and metabolism [97, 98]. During calorie restriction, AMPK and SIRT1 mutually activate one another [87, 98]. AMPK activates SIRT1 deacetylase by increasing cellular NAD + levels, thereby elevating sirtuin activity. SIRT1, in turn, facilitates AMPK phosphorylation through deacetylation of the serine–threonine kinase LKB1 [49, 87, 97, 99–101].

AMPK signaling plays a vital role in promoting longevity by enhancing health and preventing age-related diseases [97]. Activated AMPK can slow down the aging process and upregulate longevity factors such as SIRT1, P53, and forkhead box O3 (FOXO3) [102]. In rodents, metformin and resveratrol extend lifespan by enhancing AMPK and SIRT1 levels. Both compounds reduce NLRP3 and IL-1β, thereby mitigating inflammation [103–106]. Resveratrol further improves cognitive function in mouse models by activating SIRT1/AMPK signaling in alzheimer’s disease [62, 107–109]. The AMPK–SIRT1 pathway suppresses NLRP3 inflammasome activation through mitochondrial biogenesis and autophagy [20].

Activation of SIRT1, a downstream effector of AMPK, has been consistently associated with reduced NLRP3 inflammasome activity across multiple experimental models. In mouse models of brain injury, SIRT1-mediated inhibition of NLRP3 inflammasome alleviated neuronal damage, an effect reversed by SIRT1 knockdown using siRNA, confirming its role in suppressing NLRP3 activation during cerebral ischemia [90]. In line with this, pharmacological activation of SIRT1 by resveratrol suppressed NLRP3 inflammasome activation in mesenchymal stem cells, leading to reduced inflammation [110]. SIRT1 upregulation also mediated NLRP3 inhibition in various models of tissue injury, including cerebral ischemia and ischemic stroke [82, 111]. Collectively, these findings suggest that SIRT1 acts as a key negative regulator of NLRP3, potentially through coordinated regulation of mitochondrial function and autophagy. Despite these advances, the precise molecular mechanisms underlying SIRT1-mediated inhibition of the NLRP3 inflammasome remain unclear, warranting further mechanistic research to elucidate this pathway.

SIRT2 has been reported to reduce inflammation in non-alcoholic fatty liver disease by directly inhibiting the NLRP3 inflammasome [112]. SIRT3 plays a key role in maintaining mitochondrial function and redox balance. Its deletion leads to increased NLRP3 activation and mitochondrial ROS production, whereas SIRT3 activation inhibits NLRP3 and improves mitochondrial function [106]. Furthermore, suppression of SIRT3 has been linked to enhanced vascular inflammation through upregulation of the NLRP3 inflammasome [113].

### ER Stress/TXNIP Signaling Pathway

The ER is responsible for protein synthesis and folding, as well as the regulation of calcium storage and release [103, 114]. Protein folding is managed by cofactors, foldases, and chaperones [20, 115]. ER homeostasis can be disrupted by a number of factors, including physiological, environmental, and pathological stimuli; impaired calcium metabolism; oxidative stress; and elevated protein synthesis [116].

The unfolded protein response (UPR) is activated to re-establish ER homeostasis by decreasing the flow of proteins into the ER and increasing protein transfer out, thereby limiting the accumulation of unfolded proteins [20]. However, altered or sustained activation of the UPR results in ER stress, which is detected by ER transmembrane sensors such as inositol-requiring 1α (IRE1α), activating transcription factor 6 (ATF6), and protein kinase RNA-like ER kinase (PERK) [117]. Moreover, prolonged ER stress leads to cell death, inflammation, and oxidative stress [118–123]. During aging, protein clearance becomes imbalanced due to reduced autophagy function and proteasomal degradation [124]. Studies also report that UPR contributes to NF-κB activation, thereby promoting an inflammatory response [97, 119, 125, 126].

Under normal conditions, thioredoxin-interacting protein (TXNIP) binds to thioredoxin (TRX), an antioxidant, and inhibits its activity, leading to oxidative stress. Under stress, TXNIP dissociates, allowing TRX to scavenge ROS and reduce oxidative stress [117, 127–129]. ER stress-induced ROS generation triggers TXNIP detachment from TRX, facilitating TXNIP–NLRP3 binding, which activates the NLRP3 inflammasome and promotes proinflammatory cytokine release [121, 130–138]. In human and murine macrophages, ER stress activators such as thapsigargin and tunicamycin trigger NLRP3 inflammasome activation [139]. However, during starvation, ER sensor activity is reduced, thereby mitigating ER stress [103].

Activation of AMPK plays a key role in suppressing NLRP3 inflammasome activation through modulation of the TXNIP–TRX system. AMPK activation enhances TRX function, suppresses TXNIP transcription, and promotes its degradation. This prevents TXNIP from interacting with NLRP3, ultimately limiting inflammasome activation [97]. Consistent with this mechanism, natural compounds like epigallocatechin gallate, luteolin, quercetin, and mangiferin ameliorated ER stress-induced TXNIP/NLRP3 inflammasome activation via AMPK signaling, improving inflammation and apoptosis in endothelial cells [16, 118, 140].

In BV2 microglial and pancreatic cells, ROS activate TXNIP, which in turn triggers NLRP3 inflammasome activation [132, 136, 138]. Inhibiting the TXNIP/NLRP3 pathway ameliorated ischemic stroke [141] and Alzheimer’s disease-like symptoms in diabetic rats [142]. Resveratrol and berberine suppressed TXNIP/NLRP3 signaling and improved arterial function, vascular dysfunction, and MSU-induced macrophage activation [131, 143, 144].

AMPK and MAPK pathways regulate nuclear factor erythroid 2-related factor 2 (Nrf2), a key antioxidant regulator [145]. Phosphorylated AMPK facilitates Nrf2 activation [146], which enhances HO-1 expression, reduces free radicals, and lowers proinflammatory cytokines [147]. Activated Nrf2 suppresses NLRP3 inflammasome activity by modulating TXNIP and TRX expression in cerebral ischemia [148]. AMPK/Nrf2/TXNIP signaling reduced oxidative stress and neuroinflammation in the rat middle cerebral artery occlusion (MCAO) model [149]. Enhanced peroxisome proliferator-activated receptor gamma (PPARγ) expression also exerted protective effects against cerebral ischemia–reperfusion injury by inhibiting TXNIP/NLRP3 inflammasome [150].

**Fig. 2:**
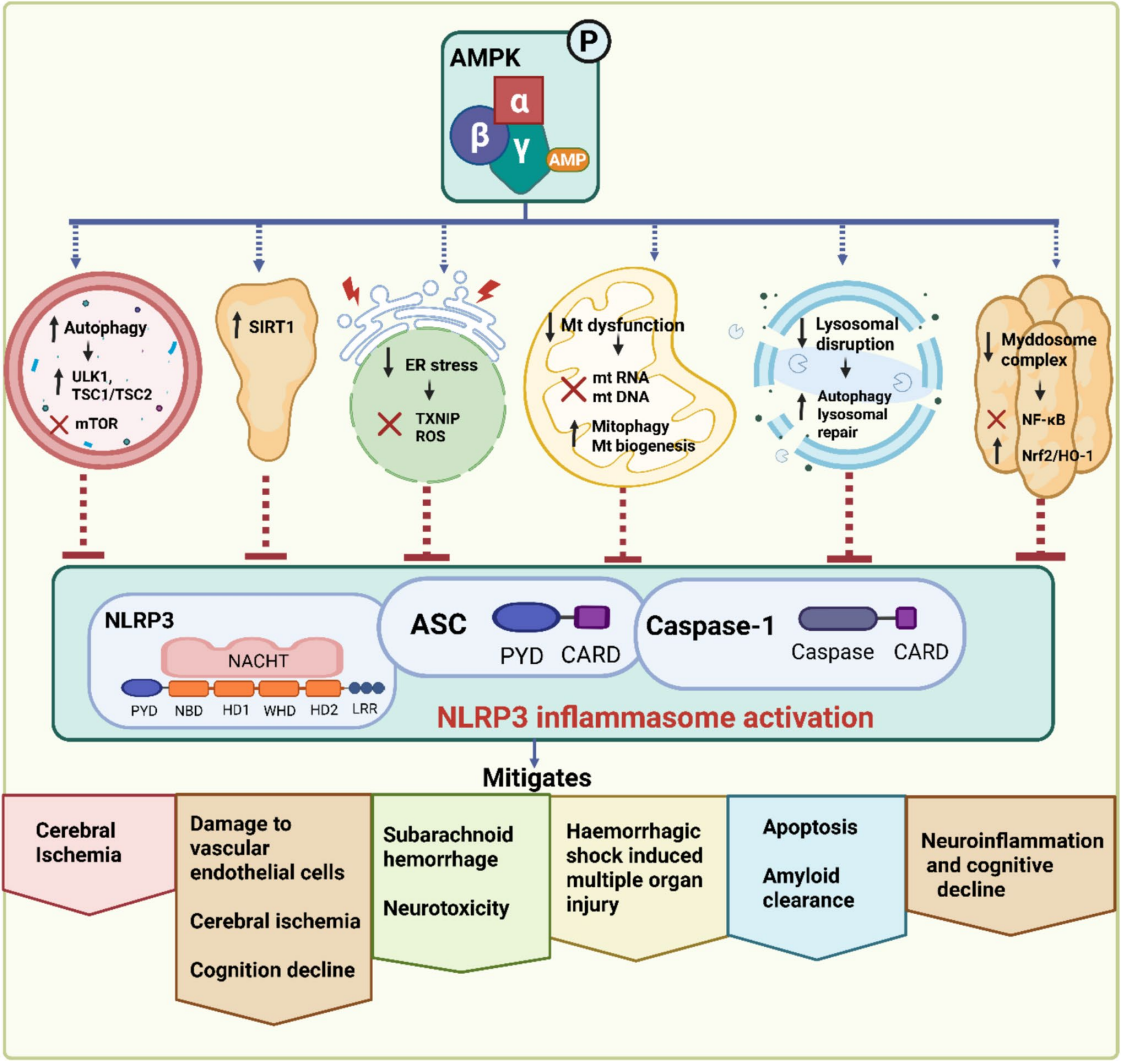
Schematic representation of mechanisms of AMPK-mediated suppression of the NLRP3 inflammasome. Phosphorylated AMPK modulates NLRP3 inflammasome activation through autophagy, sirtuins, ER stress, mitochondrial dysfunction, lysosomal disruption, and the myddosome complex, thereby mitigating NLRP3-driven diseases. AMPK, adenosine monophosphate-activated protein kinase; AMP, adenosine monophosphate; ULK1, Unc-51-like kinase 1; TSC1/TSC2, tuberous sclerosis complex 1/2; mTOR, mammalian target of rapamycin; SIRT1, silent mating type information regulation 2 homolog; ER stress, endoplasmic reticulum stress; TXNIP, thioredoxin-interacting protein; ROS, reactive oxygen species; Mt dysfunction, mitochondrial dysfunction; mtRNA, mitochondrial RNA; mtDNA, mitochondrial DNA; Mt biogenesis, mitochondrial biogenesis; NF-κB, nuclear factor kappa-light-chain-enhancer of activated B cells; Nrf2, nuclear factor erythroid 2-related factor 2; HO-1, heme oxygenase-1; NLRP3, nucleotide-binding domain leucin-rich repeat and pyrin domain-containing protein-3; NACHT, nucleotide-binding oligomerization domain; PYD, pyrin domain; NBD, nucleotide-binding domain; HD1, helical domain 1; WHD, winged helix domain; HD2, helical domain 2; LRR, leucine-rich repeat; ASC, apoptosis-associated speck-like protein; CARD, caspase-recruitment domain

Studies report that AMPK activation inhibits ER stress in various rodent models via the TXNIP/NLRP3 pathway, including glutamate-induced neurotoxicity, palmitate-induced ER stress in endothelial cells, ROS-associated ER stress, subarachnoid hemorrhage, and coronary microembolization-induced myocardial injury [17, 117, 121, 151, 152]. AMPK activators such as metformin and resveratrol ameliorate adipocyte and endothelial dysfunction by inhibiting dynamin-related protein 1 (Drp1) activity in mitochondria and preventing ER stress-induced activation of the NLRP3 inflammasome [153, 154]. Suppression of the ROS–TXNIP–NLRP3 pathway minimizes the interaction between TXNIP and NLRP3 [155, 156]. Similarly, *N*-acetyl cysteine reversed lipopolysaccharide (LPS)-induced inflammation in bone marrow mesenchymal stem cells by inhibiting TXNIP/NLRP3/IL-1β signaling [157]. Collectively, these findings demonstrate that AMPK signaling suppresses NLRP3 inflammasome activation by reducing ER stress, as illustrated in Fig. 2.

### Regulation of Mitochondrial Homeostasis

Mitochondria are vital cellular organelles that generate energy in the form of ATP, with ROS produced as a by-product of oxidative phosphorylation [158, 159]. Various stressors, including elevated metabolic demand, lack of oxygen, or mitochondrial damage, significantly increase ROS production and promote mitochondrial dysfunction [130, 160, 161]. Given their role in ROS generation, mitochondria contribute to a wide range of pathological conditions [40, 162]. Mitochondrial biogenesis must be counterbalanced with the removal of damaged mitochondria through mitophagy, a selective autophagic process that degrades dysfunctional mitochondria [163, 164]. Dysregulation of mitophagy has been linked to cell death and various neurological disorders [165, 166].

Mitochondrial homeostasis is maintained by an equilibrium balance between the fission and fusion processes. Fission is mediated by Drp1, while fusion is regulated by mitofusin-2 (Mfn2) [153]. The master regulators of mitochondrial biogenesis include PPAR-γ, PPAR-γ coactivator1α (PGC-1α), and nuclear respiratory factor-1 transcription factor (NRF1) [20, 167–169].

In response to NLRP3 stimuli, three mitochondrial membrane molecules—mitochondrial antiviral-signaling protein (MAVS; outer membrane), mitofusin-2 (outer membrane), and cardiolipin (inner membrane)—interact with NLRP3 and activate the inflammasome [2]. During viral infections (but not non-viral infections), MAVS and mitofusin-2 associate with NLRP3 and enhance inflammasome activity [170]. Under stress, cardiolipin moves to the outer membrane, where it directly interacts with NLRP3 and activates the inflammasome [171]. Increased cytosolic calcium and excessive ROS production facilitate the mitochondrial permeability transition (MPT) [172] and promote the release of oxidized mtDNA into the cytosol, which further upregulates the activation of NLRP3 inflammasome [130, 173–175]. Resveratrol attenuates MPT by reducing the release of mtDNA into the cytosol, thereby alleviating NLRP3 inflammasome and stabilizing mitochondrial function [70]. Disruption of mitochondrial complexes I and III increases mtROS, which further activates the NLRP3 inflammasome [173, 176, 177], whereas ROS inhibitors suppress the priming signal required for inflammasome activation [178]. However, excessive ROS can suppress NLRP3-mediated caspase-1 activation [179]. Hence, further research on ROS-mediated upregulation of NLRP3 activation is needed.

AMPK is activated during mitochondrial dysfunction and regulates mitophagy factors such as PTEN-induced kinase 1 (PINK), a mitochondrial kinase, and parkin, a ubiquitin ligase [165, 180]. AMPK modulates inflammasome activation by clearing damaged mitochondria through enhanced autophagy [51, 181]. In addition, AMPK attenuates mtROS-induced oxidative stress by increasing manganese superoxide dismutase (MnSOD) and catalase levels, which are critical for balancing mitophagy and mitochondrial biogenesis [182]. AMPK promotes mitochondrial biogenesis through phosphorylation of PGC-1α, a cofactor that enhances the transcription of nuclear-encoded mitochondrial genes [87, 183]. AMPK activators such as metformin and berberine have demonstrated neuroprotective effects by promoting mitochondrial biogenesis via the AMPK/PGC-1α pathway in models of cerebral ischemia and diabetic neuropathy, respectively [164, 184].

Several studies support the anti-inflammatory role of AMPK. Activation of AMPK by natural antioxidants such as γ-tocotrienol and coenzyme Q10 suppresses NLRP3 inflammasome activity [163, 185]. Enhanced mitophagy inhibits NLRP3 inflammasome-mediated pyroptosis and alleviates hemorrhagic shock-induced multi-organ injury [186]. Adiponectin suppresses NLRP3 via the AMPK–ROS pathway, whereas AMPKα1 deletion blocks this inhibitory effect in THP-1 cells [187]. Autophagy, by clearing damaged mitochondria, reduces ROS levels and inhibits NLRP3 activation [71, 188, 189]. Its disruption results in mitochondrial damage, excessive ROS production, and NLRP3 inflammasome activation [71, 173]. Targeting ROS-driven oxidative stress and the NLRP3 inflammasome pathway mitigates liver fibrosis [190].

Inhibition of mTOR by compounds like rapamycin facilitates the clearance of damaged organelles and suppresses NLRP3 inflammasome activation [76, 78]. The mTOR/NLRP3 pathway is also implicated in inflammatory responses, and its suppression mitigates intestinal inflammation [191]. In HepG2 cells and H9c2 cardiomyocytes, the AMPK/mTOR pathway has been shown to induce autophagy, offering protection against mitochondrial damage and stress [192, 193]. Activation of autophagy and inhibition of PANoptosis through mTOR promote the survival of ischemic and diabetic skin flaps [194]. Regulation of autophagy and mitophagy by the AMPK–mTOR–TFEB signaling pathway attenuated spinal cord injury [195]. Reduction in Drp1-dependent mitochondrial fission also alleviated pyroptosis-related proteins, proinflammatory cytokines, and neuroinflammation after spinal cord injury [196]. Resveratrol improved mitochondrial function and autophagy through deacetylation of PGC-1α and suppressed NLRP3 inflammasome activation [70, 197, 198]. It has also shown a protective role in preventing cancer via activation of PGC1α/SIRT1/AMPK signaling and epigenetic modulation by the DNA methyltransferase enzyme [199].

### NF-κB/MyD88-Mediated Priming Regulation

The myddosome complex plays a key role in the priming signal for NLRP3 activation and is composed of MyD88, TRIF, FADD, IRAK-1, and caspase-8. This complex is activated when PRR detect PAMPs or DAMPs, leading to NF-κB translocation into the nucleus. This process facilitates the transcription of NLRP3 and pro-IL-1β, both essential for inflammasome activation [23, 24, 200].

The MyD88-dependent NF-κB pathway is negatively regulated by AMPK activation, which reduces oxidative stress and inflammation. By activating Nrf2 and HO-1, AMPK enhances cellular defense mechanisms, counteracting the proinflammatory signaling triggered by MyD88. This represents a protective mechanism in which AMPK activation suppresses MyD88-mediated inflammation, thereby reducing neuronal damage in cerebral ischemia/reperfusion injury (CIRI) [201] and neuroinflammation [202]. Mechanistically, MyD88 contributes to the formation of the myddasome complex, which amplifies proinflammatory signaling and facilitates the priming and activation of the NLRP3 inflammasome. Consistent with this, in LPS-treated BV2 cells, activated AMPK suppressed both NLRP3 and MyD88 signaling, reducing neuroinflammation [203]. Evidence suggests that AMPK can negatively regulate NLRP3 by inhibiting the myddasome complex, thereby mitigating the inflammatory response.

### Regulation of Lysosomal Integrity

Lysosomes are the principal degradative organelles required for the efficient removal of misfolded proteins, dysfunctional organelles, and ingested pathogens [204]. Maintenance of tissue homeostasis depends on autophagic uptake and lysosomal degradation of dysfunctional organelles [205]. Lysosomal-associated membrane protein-1 (LAMP1) is the most abundant lysosomal membrane protein and serves as a marker of lysosomal stability. LAMP1 also participates in autophagy and apoptosis while preserving the structural integrity of lysosomal membranes [206].

Lysosomal membrane permeabilisation (LMP) refers to increased lysosomal membrane permeability, which aids in the release of lysosomal enzymes (cathepsin B/D) and other contents into the cytosol [207]. Released cathepsin B triggers NLRP3 inflammasome activation [206, 208]. Lysosomal disruption induces NLRP3 activation in a pH- and protease-dependent manner [209, 210]. Lysosomal cell death is thought to involve the destabilization and rupture of lysosomal vesicles [211]. Additionally, rupture of the lysosomal membrane activates NLRP3 inflammasome via the CaMKII/TAK1/JNK signaling pathway [212] and LAMP1/CaMKII/TAK1 pathway [213]. Therefore, preventing lysosomal membrane rupture may serve as a strategy to inhibit NLRP3 inflammasome activation.

In this context, AMPK plays a protective role by maintaining lysosomal function and promoting autophagy. All three subunits of AMPK are localized in lysosomes, where they contribute to autophagy [140]. AMPK reduces mTOR signaling, enhances autophagy, and promotes Aβ clearance through the lysosomal system in cell cultures [140, 214]. AMPK is activated by two possible mechanisms, depending on the duration and severity of lysosomal damage. During early stages, TAK1 activates AMPK, whereas prolonged damage increases the AMP/ATP ratio, triggering LKB1-mediated AMPK activation [215]. Collectively, these findings suggest that AMPK serves as a key regulator linking lysosomal integrity and autophagy to NLRP3 inflammasome suppression, thereby limiting inflammation under conditions of cellular stress.

## AMPK-Mediated Suppression of the NLRP3 Inflammasome in CNS-Related Disease

**Fig. 3:**
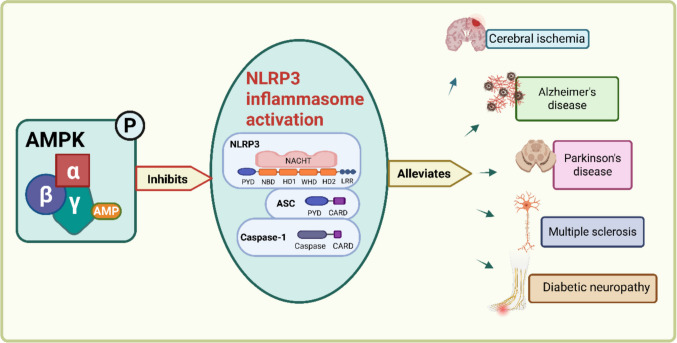
AMPK-mediated suppression of the NLRP3 inflammasome in CNS-related diseases. Phosphorylated AMPK inhibits NLRP3 inflammasome activation and alleviates NLRP3-mediated conditions such as cerebral ischemia, Alzheimer’s disease, Parkinson’s disease, multiple sclerosis, and diabetic neuropathy. AMPK, adenosine monophosphate-activated protein kinase; AMP, adenosine monophosphate; NLRP3, nucleotide-binding domain leucin-rich repeat and pyrin domain-containing protein-3; NACHT, nucleotide-binding oligomerization domain; PYD, pyrin domain; NBD, nucleotide-binding domain; HD1, helical domain 1; WHD, winged helix domain; HD2, helical domain 2; LRR, leucine-rich repeat; ASC, apoptosis-associated speck-like protein; CARD, caspase-recruitment domain

AMPK is a key immunometabolic regulator of neuroinflammation through modulation of the NLRP3 inflammasome. AMPK activation consistently inhibits NLRP3 in several neuroinflammatory conditions, including ischemic stroke, Alzheimer’s disease, Parkinson’s disease, multiple sclerosis, and diabetic neuropathy. This is discussed in detail below (Fig. 3).

### Ischemic Stroke/Cerebral Ischemia

In a mouse model of MCAO-induced cerebral ischemia, as well as an in vitro model of oxygen–glucose deprivation (OGD) in astrocytes and microglia, NLRP3 inflammasome activation correlates with reduced AMPK phosphorylation, thereby increasing inflammation. Suppression of AMPK further enhances NLRP3 activation, worsening post-ischemic inflammatory injury. Conversely, activation of AMPK provides protection against ischemic stroke by inhibiting NLRP3 inflammasome activity in both in vitro and in vivo models [216], as illustrated in Fig. 3.

In the rat MCAO model, inhibition of the NLRP3 inflammasome combined with activation of AMPK signaling attenuates cerebral infarction, BBB permeability, brain edema, and neuronal apoptosis [40]. Studies indicate that suppressing NLRP3 reduces neurovascular damage, brain injury, and inflammation in the MACO model [217–219]. AMPK activation may serve as an early protective mechanism in cerebral ischemia [220], highlighting the therapeutic potential of targeting the AMPK/NLRP3 pathway.

### Alzheimer’s Disease

Activation of AMPK signaling promotes mitophagy through ULK1 and PINK1/parkin pathways, thereby restoring mitochondrial function. Concurrently, suppresses NLRP3 inflammasome-mediated microglial pyroptosis and neuroinflammation. This AMPK–NLRP3 modulation reduces Aβ pathology and supports neuronal integrity, contributing to improved cognitive function. CAMKK2-mediated AMPK phosphorylation further enhances autophagy in astrocytes. AMPK-driven autophagy promotes Aβ clearance and suppresses NLRP3 inflammasome activation [221].

Activation of the SIRT1/AMPK/mTOR pathway enhances autophagy, restores mitochondrial homeostasis, and reduces mtROS. This AMPK-driven effect suppresses NLRP3 inflammasome activation, thereby attenuating neuroinflammation and improving cognitive function in Aβ₁₋₄₂-induced models [222].

### Parkinson’s Disease

AMPK activation suppresses Drp1-mediated mitochondrial fragmentation and restores mitochondrial function. AMPK-driven mitochondrial stabilization reduces mtROS, thereby inhibiting NLRP3 inflammasome activation in neurons. Activation of the AMPK/SIRT1 signaling pathway also suppresses NLRP3 inflammasome components (NLRP3, caspase-1, IL-1β), reducing neuroinflammation in the prefrontal cortex. This AMPK–NLRP3 axis improves behavioral outcomes, attenuates neuroinflammation and dopaminergic neuronal loss, and contributes to neuroprotection in rotenone-induced models and 6-OHDA-induced Parkinson’s disease [223, 224].

### Multiple Sclerosis

Activation of the AMPK/SIRT1 pathway enhances autophagy and inhibits TLR4/NF-κB-mediated NLRP3 inflammasome activation. This coordinated regulation of AMPK and NLRP3 pathways attenuates neuroinflammation and cuprizone-induced demyelination in C57Bl/6 mice [225]. Inhibition of HDAC3 promotes AMPK-mediated mitophagy and inhibits NLRP3 activation, leading to reduced microglial activation, neuroinflammation, and demyelination in both BV2 cells and the hippocampus of EAE mice [226].

### Diabetic Neuropathy

AMPK activation in dorsal root ganglia suppresses NLRP3 inflammasome activation and improves insulin sensitivity, thereby modulating the AMPK–NLRP3 axis under hyperglycemic conditions. This regulation attenuates neuroinflammation and alleviates diabetic neuropathic pain, highlighting AMPK-dependent inhibition of NLRP3 as a key mechanism.

Activation of the AMPK/SIRT1/PGC-1α pathway improves mitochondrial function and cellular energy homeostasis. Additionally, AMPK activation concurrently suppresses the GPR40/β-arrestin2-mediated NLRP3 inflammasome pathway, reducing inflammatory responses [227]. A study demonstrated that activated AMPK enhances mitochondrial quality control through autophagy and biogenesis (PINK1/parkin, Nrf2 pathways). Additionally, it reduces oxidative stress and suppresses NF-κB-mediated NLRP3 inflammasome activation. This AMPK–NLRP3 interplay alleviates neuroinflammation, mitigates neuropathic pain, and improves Schwann cell function and mitochondrial function [18].

## AMPK and NLRP3 Interplay in Neuroinflammation Models

Neuroinflammation is the root cause of most neurodegenerative disorders, such as Alzheimer’s disease, Parkinson’s disease, schizophrenia, cognitive impairment, and even cancers of the central nervous system [228]. NLRP3 plays a role in the pathophysiology of different neuronal and metabolic disorders [229]. In the CNS, the NLRP3 inflammasome is abundantly expressed and sometimes acts as a noxious agent that dysregulates the cellular microenvironment [230]. Targeting the NLRP3 inflammasome to mitigate inflammatory responses represents a promising strategy for managing neurodegenerative disorders. However, research in this area is still in its early stages. Evidence suggests that activation of AMPK signaling promotes anti-inflammatory and neuroprotective effects. Therefore, exploring the AMPK–NLRP3 signaling in neuroinflammation provides a novel therapeutic strategy for managing neurodegenerative disorders, opening new avenues for drug development and intervention. The targeted pathways and experimental outcomes of neuroinflammatory models, such as traumatic brain injury (TBI), streptozotocin (STZ), and LPS-induced neuroinflammation, are listed in Table 1.

**Table 1:** Regulation of NLRP3 by AMPK in neuroinflammatory models

Sr. No	Disease model	Targeted pathways	Experiment outcomes	Reference
1	TBI-induced acute cerebral damage in C57BL/6 J mice and oxygen–glucose deprivation (OGD) in BV2 microglial cells	AMPK/SIRT1/FoxO1/NF-κB/NLRP3 inflammasome	Ameliorated neuronal apoptosis, cerebral edema, neurological deficits, microglial activation, and proinflammatory cytokine production	[231]
2	TBI in C57BL/6 mice (wild type, *n* = 32; RIP3-knockout, *n* = 32) and LPS-treated astrocytes	AMPK/NF-κB/NLRP3 inflammasome	Reduced apoptosis, inflammation, and oxidative stress, with improved cognition	[232]
3	TBI in BALB/c mice	AMPK/mTOR/NLRP3 inflammasome	Improved neurological recovery and enhanced neuronal autophagy	[233]
4	STZ-induced DN in Sprague–Dawley (SD) rats and high-glucose-induced hyperglycemia in Schwann cells	AMPK/NFκB/sirT3/Nrf2/NLRP3 inflammasome	Mitigated neuronal injury and neuroinflammation	[18]
5	MACO-induced acute cerebral ischemia in C57BL/6 mice and LPS-activated BV2 microglial cells	AMPK/NLRP3 protein expression	Alleviated ROS‑induced oxidative stress and neuroinflammation	[234]
6	MACO-induced cerebral ischemia in SD rats and LPS-induced activation of BV2 microglial cells	AMPK/mTOR/ULK1/NLRP3 inflammasome	Enhanced autophagy, reduced microglial activation, and improved neuronal deficits	[235]
7	LPS-induced depression model in SD rats and LPS-treated BV2 microglia	AMPK/NLRP3 inflammasome	Promoted autophagy, ameliorated neuroinflammation, and improved behavioral deficits	[236]
8	Stefin B trisomic mice BMDMs and RAW264.7 macrophages following LPS stimulation	AMPK/mTOR/NLRP3 inflammasome	Mitigated mitochondrial ROS generation, reduced inflammatory response, and enhanced autophagy	[19]
9	5 × FAD mice and LPS-primed microglia	AMPK/Syk/NLRP3 inflammasome	Mitigated cognitive dysfunction	[237]

## Therapeutic Targeting of the AMPK–NLRP3 Axis in Neuroinflammatory and Neurodegenerative Disorders

Emerging evidence suggests that the inhibitory effects of AMPK on NLRP3 inflammasome activation are mediated not by a single downstream pathway but rather by the convergence of multiple regulatory processes, including restoration of energy homeostasis, enhancement of autophagic clearance, reduction of mitochondrial dysfunction and ROS generation, and modulation of stress- and inflammation-associated signaling pathways. Together, these coordinated actions may underlie the therapeutic potential of AMPK activation in neuroinflammatory and neurodegenerative disorders.

### AMPK Activators with Neuroprotective Effects

Pharmacological activation of AMPK has emerged as a promising strategy to modulate neuroinflammation and metabolic dysfunction. AMPK activators, including metformin and resveratrol, have demonstrated neuroprotective effects in experimental models by enhancing mitochondrial function, promoting autophagy/mitophagy, and reducing oxidative stress, ultimately suppressing NLRP3 inflammasome activation and downstream inflammatory signaling, thereby improving neuronal survival and functional outcomes in experimental models [238, 239].

AICAR treatment activates AMPK, downregulates TXNIP, disrupts TXNIP-NLRP3 binding, and reduces NLRP3 inflammasome activation in rat ischemia–reperfusion-induced acute kidney injury [240]. Additionally, AICAR ameliorates oxidative stress and inflammation by increasing *p*-AMPK and Nrf2 and inhibits NLRP3 inflammasome activation in the liver tissues of sodium taurocholate-induced severe acute pancreatitis rats [241]. There is no direct evidence that AICAR attenuates neuroinflammation by modulating the AMPK–NLRP3 pathway. Although direct evidence in CNS-specific models is limited, this mechanism suggests that AICAR-mediated AMPK activation could plausibly reduce microglial activation and neuroinflammation through suppression of TXNIP-dependent NLRP3 signaling.

Similarly, salicylate activates AMPK and suppresses TXNIP expression, thereby inhibiting NLRP3 inflammasome assembly in vascular and metabolic models [154]. Although direct evidence remains limited, AMPK activation is a well-established negative regulator of NLRP3-driven inflammasome signaling in microglia, suggesting that salicylate-mediated AMPK activation can plausibly suppress NLRP3-related neuroinflammation.

### Direct Inhibitors of NLRP3 Inflammasome

Targeting NLRP3 directly is another therapeutic approach. Small molecule inhibitors such as MCC950, CY-09, JC-171, OLT1177, oridonin, and tranilast effectively block NLRP3 activation and decrease caspase-1 activity, leading to reduced IL-1β/IL-18 release and, consequently, decreased neuroinflammation and slowed disease progression in preclinical models of neurodegenerative disorders [23]. However, clinical translation remains challenging, as phase II clinical trials of MCC950 in rheumatoid arthritis were discontinued due to hepatotoxicity [242].

Although direct NLRP3 inhibitors have shown promising anti-inflammatory effects, targeting AMPK offers a broader therapeutic advantage by acting upstream to modulate mitochondrial dysfunction, oxidative stress, and autophagy, thereby preventing inflammasome activation while simultaneously improving cellular metabolic homeostasis. This multifaceted regulation makes AMPK a more comprehensive and translationally relevant target in neuroinflammatory and neurodegenerative diseases.

### Natural Compounds and Metabolic Modulators Affecting Both AMPK Signaling and NLRP3 Activation in Neuroinflammation

Metformin activates AMPK and inhibits NLRP3 inflammasome activation, resulting in reduced brain edema, behavioral disorders, cell apoptosis, and neuronal injury in subarachnoid hemorrhage [238]. Similarly, compounds such as quercetin, resveratrol, AICAR, and berberine modulate AMPK signaling and suppress NLRP3 activation in peripheral tissues. However, evidence supporting this mechanistic interplay within the CNS remains limited. Therefore, further studies are required to elucidate the AMPK–NLRP3 regulatory axis in CNS-specific contexts, particularly considering factors such as BBB permeability, cell-type specificity, and the neuroinflammatory microenvironment.

### Translational Considerations for Neurological Therapy

Despite promising preclinical findings, several challenges must be addressed for clinical translation. Effective therapeutic agents must demonstrate adequate BBB penetration, high pharmacological specificity, and minimal off-target effects. Additionally, variability in disease pathology and patient response necessitates careful evaluation in clinical trials. Although some AMPK activators, such as metformin, are already clinically approved for other indications, NLRP3 inhibitors are still largely in preclinical or early clinical development, highlighting the need for further translational research. In this context, repurposing clinically approved AMPK activators offers a promising strategy for CNS disorders, as their ability to modulate the AMPK–NLRP3 axis, combined with established safety profiles, may facilitate more rapid clinical translation.

## Future Directions and Conclusion

The NLRP3 inflammasome plays a key role in inflammation, requiring both priming and activation signals for its activation [23]. AMPK plays a central role in regulating innate immunity and inflammation by enhancing autophagy, maintaining mitochondrial homeostasis, regulating ER stress, stabilizing lysosomes, activating SIRT1, and suppressing NLRP3 inflammasome activity [20]. Investigating AMPK’s role in inflammasome priming and activation across disease models is crucial. Targeting AMPK provides a dual benefit by inhibiting NLRP3 inflammasome activation and broadly enhancing cellular health through improved energy balance and reduced oxidative stress. Future drug development should prioritize the discovery of selective AMPK activators with enhanced bioavailability and specificity to effectively suppress NLRP3 activation while minimizing off-target effects.

Currently, there are no FDA-approved drugs for AMPK activation or NLRP3-specific inhibitors for CNS diseases or neuroinflammation. Certain approved drugs for other conditions have shown potential benefits in AMPK activation and indirect inhibition of NLRP3 activation, primarily through off-label use or ongoing research. For instance, metformin, an anti-diabetic drug, activates AMPK and has shown neuroprotective effects in animal studies of neurodegeneration [243, 244]. Mefloquine, an antimalarial drug, has been identified as a potent NLRP3 inhibitor in preclinical studies, showing potential in reducing neuroinflammation [245].

Additionally, integrating pharmacological interventions with lifestyle modifications, such as exercise and dietary strategies, could offer a holistic approach to controlling NLRP3-driven inflammation. Exploring the potential benefits of combining AMPK activators with known NLRP3 inhibitors to assess synergistic effects in preclinical and clinical models of conditions such as neurodegenerative diseases, metabolic disorders, and autoimmune diseases. Although NLRP3 inhibitors such as MCC950 have shown promise in preclinical studies, their long-term safety and efficacy in human clinical trials remain a major challenge. Further research should explore bioactive compounds that boost AMPK activity and suppress NLRP3, offering potential treatments for NLRP3-driven inflammatory diseases.

Variability in experimental models, disease conditions, and AMPK activators may affect reproducibility. Cell-specific and context-dependent effects of AMPK signaling are not fully understood. The crosstalk between AMPK and other signaling pathways also complicates mechanistic interpretation.

Nevertheless, current evidence largely relies on in vitro and animal studies, and direct molecular mechanisms underlying AMPK-mediated regulation of the NLRP3 inflammasome remain incompletely understood. Therefore, further mechanistic and clinical studies are necessary to establish translational relevance.

Collectively, targeting the AMPK–NLRP3 axis represents a promising and multifaceted clinical strategy, offering potential benefits in modulating neuroinflammation, restoring metabolic homeostasis, and slowing the progression of neurodegenerative and neuroinflammatory disorders.

## Data Availability

No datasets were generated or analysed during the current study.

## Abbreviations

NLRP3: Nucleotide-binding domain leucine-rich repeat and pyrin domain-containing protein-3
AMPK: Adenosine monophosphate-activated protein kinase
CNS: Central nervous system
PYD: N-terminal pyrin domain
NACHT: Nucleotide-binding and oligomerization domain
LRR: Leucine-rich repeat
NBD: Nucleotide-binding oligomerization domain
WHD: Winged helix domain
HD2: Helical domain 2
PAMPs: Pathogen-associated molecular patterns
DAMPs: Damage-associated molecular patterns
KD: Kinase domain
LKB1: Liver kinase B1
TAK1: Transforming growth factor β-activated kinase 1
AID: Autoinhibitory domain
CTD: C-terminal domain
CBM: Carbohydrate-binding module
ADaM: Allosteric drug and metabolite
MCAO: Middle cerebral artery occlusion
OGD: Oxygen-glucose deprivation
ALI: Acute liver injury
DN: Diabetic nephropathy
LPS: Lipopolysaccharide
SLE: Systemic lupus erythematosus
BECN-1: Beclin-1
ULK1: Unc-51-like kinase 1, a mammalian ortholog of Atg1
TSC2: Tuberous sclerosis protein 2
ATG9: Autophagy-related 9 proteins
mTOR: Mammalian target of rapamycin
TXNIP: Thioredoxin-interacting protein
PPAR-γ: Peroxisome proliferator-activated receptor
PGC-1α: PPAR-γ coactivator1α
MAVS: Mitochondrial antiviral-signaling protein
MPT: Mitochondrial permeability transition
LAMP1: Lysosomal-associated membrane protein-1
TBI: Traumatic brain injury
STZ: Streptozotocin
